# Epidemiology of Leprosy in Iran from 2005 to 2015

**Published:** 2017

**Authors:** Kamyar Mansori, Erfan Ayubi, Mahshid Nasehi, Shiva Mansouri Hanis, Behzad Amiri, Salman Khazaei

**Affiliations:** 1 Social Development and Health Promotion Research Center, Gonabad University of Medical Sciences, Gonabad, Iran,; 2 Department of Epidemiology, School of Public Health, Iran University of Medical Sciences, Tehran, Iran,; 3 Department of Community Medicine, School of Medicine, Zahedan University of Medical Sciences, Zahedan, Iran,; 4 Dezful University of Medical Sciences, Dezful, Iran,; 5 Centers for Communicable Disease Control and Prevention, Ministry of Health and Medical Education, Tehran, Iran,; 6 Department of Epidemiology, School of Public Health, Hamadan University of Medical Sciences, Hamadan, Iran,; 7 Department of Epidemiology and Biostatistics, School of Public Health, Tehran University of Medical Sciences, Tehran, Iran.

**Keywords:** Epidemiologic study, Trend, Leprosy, Iran

## Abstract

**Background:**

Leprosy is a chronic infectious disease with permanent complications that mainly affect the skin, peripheral nerves, mucosal surfaces of the upper respiratory tract, and eyes. The aim of this study was to investigate the epidemiology and trends of leprosy in Iran from 2005 to 2015.

**Materials and Methods:**

This was a cross-sectional study analyzing leprosy records from the Center for Communicable Disease Control, Ministry of Health and Medical Education, during 2005–2015.

**Results:**

Of the 433 cases of leprosy diagnosed from 2005 to 2014, 87.1% were Iranian, and 56.2% of the Iranian cases were male. Furthermore, 82.5% of cases were multibacillary. The paucibacillary leprosy cases had a better remission rate in most years of the study. The annual prevalence and case detection rates of leprosy (per 100,000 population) significantly decreased in Iran between 2005 and 2015: from 0.2 to 0.02 and from 0.11 in 2005 to 0.02, respectively. The geographical distribution of leprosy cases in 2014 showed that leprosy is more common in the west, north, northwest, and south of Iran.

**Conclusion:**

Although Iran is currently an area in which leprosy is not a serious problem, new cases of leprosy are still diagnosed in Iran. Considering that Iran is attempting to eradicate the disease, careful attention to all aspects of the disease is essential.

## INTRODUCTION

Leprosy, or Hansen’s disease, is a chronic infectious disease with permanent complications that mainly affect the skin, peripheral nerves, mucosal surfaces of the upper respiratory tract, and eyes. Leprosy is caused by *Mycobacterium leprae* and can occur at all ages, ranging from early infancy to very old age (1). Leprosy can be classified on the basis of clinical manifestations and skin smear results. In the classification based on skin smears, patients showing negative smears at all sites are said to have paucibacillary leprosy, while those showing positive smears at any site are said to have multibacillary leprosy (2).

Despite the global prevalence, new information shows a drop in the number of cases of this disease. In 1992, there were 5.5 million leprosy patients in the world, but there were only 1,150,000 and 612,110 new cases of leprosy in 1997 and 2002, respectively (3). In 2015, the global prevalence of leprosy was 176,176 cases (0.2 cases per 10,000 people) with 211,973 new cases (2.9 new cases per 100,000 people) according to reports from 138 countries in all World Health Organization regions. There were 215,656 and 213,899 new cases of leprosy in 2013 and 2014, respectively. Global statistics imply that 94% of new leprosy cases are related to 14 countries with more than 1000 new cases each and only 6% of new cases are related to the rest of the world (4). India has the greatest number of cases with 59%, followed by Brazil and Indonesia with 14% and 8%, respectively (5).

The most important risk factor for developing leprosy is contact with another person with leprosy, which increases the likelihood of developing leprosy 5 to 8 times. Other risk factors including poor health, conditions related to reduced immune function such as age, malnutrition, health status, and previous exposure to other illnesses, or host genetic differences may increase the risk of developing leprosy (6).

Leprosy is well known as a disease of poverty; indeed, it is endemic in the poorest countries of the world, and socioeconomic determinants have been shown to be a major influence on the continuing transmission of leprosy (7). The sex ratios of leprosy patients being diagnosed and treated are dependent on regional differences. In Asia, the disease is more common in men than women, while in Africa, it is more common in women (8). In terms of disease transmission, nasal droplets of untreated patients are the main source of infection, whereas the skin does not have much of a role in the transmission of leprosy (9, 10).

Although the Middle East and Iran are currently areas in which leprosy is not a serious problem, new cases of leprosy are still diagnosed in Iran. However, given that Iran is trying to eradicate the disease, careful attention to all aspects of the disease is essential. Therefore, the aim of this study was to investigate the epidemiology and trends of leprosy in Iran from 2005 to 2015.

## MATERIALS AND METHODS

This was a cross-sectional study analyzing leprosy records from the Center for Communicable Disease Control, Ministry of Health and Medical Education, during 2005–2015 (11).

The diagnosis of leprosy is based on clinical signs and symptoms and, in some situations, laboratory and skin culture results were used to identify cases. In endemic areas, subjects with one of the following signs were considered to have leprosy: skin lesion consistent with leprosy and definite sensory loss with or without thickened nerves and positive skin smears (1). In Iran, case finding, treatment, follow-up, recording, and reporting for the leprosy elimination program are conducted through a healthcare network. The leprosy program has been integrated into the primary healthcare system since 1991.

Descriptive analysis including tables, charts, and graphs are used for presenting and summarizing the data. For detecting changes in trends of the prevalence and detection rates of leprosy per 100,000 population, the Cochran-Armitage test was used, and a p-value<0.05 was considered significant for all statistical tests. Stata software version 12 (Stata Corp., College Station, TX, USA) was used for all analytical operations.

## RESULTS

Of 433 leprosy cases detected from 2005 to 2014, 377 (87.1%) were Iranian, and 212 (56.2%) of the Iranian cases were male. In all years except 2012, 2013, and 2015, there were more male cases than female cases (Table 1).

**Table 1.** Trend of the frequency of detected leprosy cases in Iran by nationality and gender (2005–2015)

**Year**	**Frequency**	**Nationality (%)**		**Gender (Iranian cases)**		

		**Iranian**	**Afghan**	**Male**	**Female**	**Male/Female ratio**
2005	90	83 (92)	7 (8)	44	39	1.12
2006	72	65 (90)	7 (10)	34	31	1.10
2007	41	37 (90)	4 (10)	21	16	1.31
2008	38	32 (84)	6 (16)	18	14	1.29
2009	48	38 (79)	10 (21)	23	15	1.53
2010	35	31 (89)	4 (9)	22	9	2.44
2011	28	25 (89)	3 (11)	16	9	1.78
2012	26	25 (96)	1 (4)	12	13	0.92
2013	16	13 (81)	3 (13)	6	7	0.86
2014	26	20 (77)	6 (23)	12	8	1.50
2015	13	8 (62)	5 (39)	4	4	1.00
Total	433	377 (87.1)	56 (12.9)	212 (56.2)	165 (43.8)	-

During the study period, 311 cases (82.5%) were multibacillary, and with the exceptions of 2005 and 2007, more than 85% of cases were multibacillary in other years. In most years, the paucibacillary leprosy cases had a better remission rate. We found no distinct trend for remission rate throughout the study period (Table 2).

**Table 2.** Trend of the frequency of detected leprosy cases in Iran by Leprosy type, remission rate and history of close contact with positive case

**Year**	**Leprosy type N (%)**		**Remission rate (%)**		**History of close contact with positive case (%)**	

	**Multibacilli**	**Paucibacillary**	**Multibacilli**	**Paucibacillary**	**Yes**	**No**
2005	48 (58)	35 (42)	44	50	30	70
2006	59 (91)	6 (9)	55	91	9	91
2007	29 (78)	8 (22)	72	83	8	92
2008	29 (91)	3 (9)	55	100	9	91
2009	33 (87)	5 (13)	69	100	13	87
2010	29 (93)	2 (7)	79	100	16	84
2011	22 (88)	3 (12)	76	50	4	96
2012	22 (88)	3 (12)	87.5	100	20	80
2013	13 (100)	0	91	75	8	92
2014	20 (100)	0	92	-	0	100
2015	7 (88)	1 (12)	81	-	38	62
Total	311(82.5)	66 (17.5)	-	-	-	-

**Figure 1.**
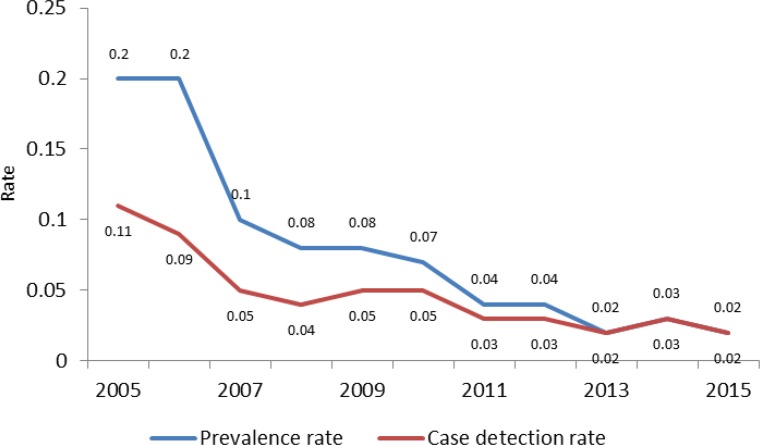
Trend of leposy prevalence rate an case detection rate (per 100,000) in Iran (2005–2015)

According to Figure 1, the annual prevalence rate and case detection rate (per 100,000) for leprosy in Iran progressively declined between 2005 and 2015 (*P*_trend_<0.001): from 0.2 to 0.02 and from 0.11 to 0.02, respectively.

**Figure 2.**
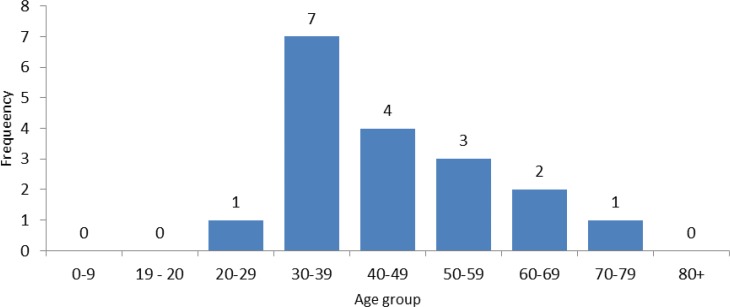
Frequency of Iranian leprosy cases by age group, 2014

The frequencies of Iranian leprosy cases by age group in 2014 are shown Figure 2, with all cases being more than 20 years of age. Of 18 detected cases in 2014, 7 (38.9%) were in the 30- to 39-year age group.

Geographical distribution of the cases in 2014 (Figure 3) showed that leprosy occurred most frequently in the west, north, northwest, and south of Iran. Golestan, West Azerbaijan, and Zanjan Provinces reported higher prevalence rates of leprosy in 2014 compared with other provinces.

## DISCUSSION

The aim of this study was to investigate the epidemiology and trends of leprosy in Iran from 2005 to 2015. The present research was a cross-sectional study analyzing leprosy records from the Center for Communicable Disease Control, Ministry of Health and Medical Education, during 2005–2015.

The results of this study showed that of the total cases of leprosy diagnosed from 2005 to 2014, 87.1% were Iranian, and 56.2% of the Iranian cases were male. Furthermore, 82.5% of the cases were multibacillary and 17.5% were paucibacillary. The cases of paucibacillary leprosy in most years under study had a better remission rate. The annual prevalence rate and case detection rate of leprosy significantly decreased in Iran so that the prevalence rate of 0.2 in 2005 decreased to 0.02 per 100,000 in 2015, and the case detection rate of 0.11 in 2005 decreased to 0.02 per 100,000 in 2015. All cases of leprosy diagnosed in 2014 were older than 20 years and more they were in the age group of 30–39 years. The geographical distribution of the cases in 2014 showed that leprosy is more common in the west, north, northwest, and south of Iran, and Golestan, West Azerbaijan, and Zanjan Provinces had the highest prevalence rates of leprosy in 2014.

In our study, from 2005 to 2015, the majority of diagnosed cases of leprosy (87.1%) were Iranian, and 12.9% of them were Afghan refugees. Although the total number of cases of leprosy was low in Afghan refugees during this period, in 2015, they accounted for 39% of leprosy cases. That is a significant percentage, because given that Iran is currently at the elimination stage of this disease (defined as a prevalence of less than 1 case per 10,000 population), the occurrence of such cases in the Afghan refugees can be an obstacle for achieving eradication in Iran. Therefore, in prevention and control programs for leprosy, particular attention of healthcare systems to Afghan refugees is essential.

In this study, we identified 377 patients with lepromatous disease of whom 212 (56.2%) were male and 165 (43.8%) were female, and except for 2012, 2013, and 2015, there were more male than female cases each year. This result was consistent with findings of other studies conducted in Iran and other countries (3, 12–14). For example, Golfurushan and colleagues reported that of 195 new cases of leprosy in Azerbaijan, Iran (from 1994 to 2009), 131 patients (67.2%) were male (13).

In our study, 82.5% of diagnosed cases of leprosy were multibacillary and 17.5% were paucibacillary, which had a better remission rate in most years. These observations were similar to results of other studies conducted in this field (11, 13, 14). In the research conducted by Toweir and Chaudhary in Benghazi, the ratio of multibacillary to paucibacillary cases was 1.3:1 (15). However, this observation is not consistent with the study of Santos et al. who found that of 1265 cases of leprosy, 933 (73.8%) were paucibacillary and 332 (26.2%) were multibacillary (16). Given that the majority of diagnosed cases of leprosy were multibacillary in Iran and that the possibility of transmission and severity of disease are higher in this type of leprosy than in paucibacillary leprosy, it is essential that intervention programs of prevention and treatment are focused on these patients.

In the present study, the annual prevalence rate and case detection rate of leprosy had significantly decreased in Iran so that the prevalence rate of 0.2 in 2005 decreased to 0.02 per 100,000 in 2015, and the case detection rate of 0.11 in 2005 decreased to 0.02 per 100,000 in 2015. These results are consistent with other reports and studies (3, 5, 6, 13). In the investigation conducted by Schreuder and colleagues to describe epidemiologic trends for the 21st century, the prevalence rate and case detection rate of leprosy significantly decreased worldwide (6).

In this study, all cases of leprosy were diagnosed in patients older than 20 years of age in 2014 and more they were in age group of 30–39 years, which is similar to the study by Golfurushan et al. showing the mean age of diagnosed cases of leprosy in women and men were 42 and 38 years, respectively (13). This result is also consistent with other studies (3). In our study, the geographical distribution of the cases in 2014 showed that leprosy is more common in the west, north, northwest, and south of Iran, and Golestan, West Azerbaijan, and Zanjan Provinces had the highest prevalence rate of leprosy in 2014. The different reports of the Center for Communicable Disease Control, Ministry of Health and Medical Education of Iran, shows that these regions have a higher prevalence of leprosy than other regions of Iran (11). Therefore, in the eradication program of leprosy, considering this age group and these high-risk regions are especially important.

## CONCLUSION

Although leprosy is not currently a serious problem in the Middle East and Iran, new cases of leprosy are still diagnosed in Iran. Given that Iran is trying to eradicate leprosy, careful attention to all aspects of this disease is essential.

## References

[1] Eichelmann K González SG Salas-Alanis JC Ocampo-Candiani J Leprosy. An update: definition, pathogenesis, classification, diagnosis, and treatment. Actas Dermo-Sifiliográficas (English Edition) 2013;104(7):554–63. 10.1016/j.adengl.2012.03.028 23870850

[2] Torres P Camarena JJ Gomez JR Nogueira JM Gimeno V Navarro JC Comparison of PCR mediated amplification of DNA and the classical methods for detection of Mycobacterium leprae in different types of clinical samples in leprosy patients and contacts. Lepr Rev 2003;74(1):18–30. 12669929

[3] Kavuossi H Najafi F Janbakhsh A Ghadiri K Epidemiology of leprosy in Kermanshah province from 2003 to 2008. Journal of Kermanshah University of Medical Sciences (J Kermanshah Univ Med Sci) 2010;13(4).

[4] Nourdin SK A guidline to eliminating leprosy as a public health problem. Majdi Sh Angizeh A (In Persian). 2th ed Tehran; Sadra publication 1997:9–16.

[5] World Health Organization Leprosy elimination [cited Dec 2016]; Available from: http://www.who.int/lep/epidemiology/en/.

[6] Schreuder PA Noto S Richardus JH Epidemiologic trends of leprosy for the 21st century. Clin Dermatol 2016;34(1):24–31. 26773620 10.1016/j.clindermatol.2015.11.001

[7] Feenstra SG Nahar Q Pahan D Oskam L Richardus JH Social contact patterns and leprosy disease: a case-control study in Bangladesh. Epidemiol Infect 2013;141(3):573–81. 22583511 10.1017/S0950268812000969 PMC9167658

[8] Varkevisser CM Lever P Alubo O Burathoki K Idawani C Moreira TM Gender and leprosy: case studies in Indonesia, Nigeria, Nepal and Brazil. Lepr Rev 2009;80(1):65–76. 19472853

[9] Chehl S Job CK Hastings RC Transmission of leprosy in nude mice. Am J Trop Med Hyg 1985;34(6):1161–6. 3914846 10.4269/ajtmh.1985.34.1161

[10] Pedlejc Composite skin contact smears: a method of demonstrating the non-emergence of Mycobacterium leprae from intact lepromatous skin. Lepr Rev 1970;41(1):31–43. 4909827

[11] Gooya MM Nasehi M Moghaddam EM Leprosy Situation In I.R.IRAN 2015: Center for Communicable Disease Control, Ministry of Health and Medical Education; 2016.

[12] Boggild AK Keystone JS Kain KC Leprosy: a primer for Canadian physicians. CMAJ 2004;170(1):71–8. 14707226 PMC305320

[13] Golfurushan F Sadeghi M Goldust M Yosefi N Leprosy in Iran: an analysis of 195 cases from 1994–2009. J Pak Med Assoc 2011;61(6):558–61. 22204210

[14] Golforoushan F Razi A Azimi H Herischi H Review of Tenyrs Leprosy Cases in Azerbaijan, Northwest of Iran. Iranian Journal of Medical Sciences 2006;31(2):103–5.

[15] Toweir AA Chaudhary RC Review of leprosy cases in Benghazi, Libyan Arab Jamahiriya, 1994–1998. Eastern Mediterranean Health Journal 2000; 6(5): 1098–102. 12197333

[16] Santos VS de Mendonça Neto PT Falcão Raposo OF Fakhouri R Reis FP Feitosa VL Evaluation of agreement between clinical and histopathological data for classifying leprosy. Int J Infect Dis 2013;17(3):e189–92. 23158973 10.1016/j.ijid.2012.10.003

